# Endogenous Losses of Fat and Fatty Acids in Growing Pigs Are Not Affected by Vegetable Oil Sources but by the Method of Estimation

**DOI:** 10.3390/ani10010048

**Published:** 2019-12-25

**Authors:** Lu Wang, Li Wang, Zhiqian Lyu, Bingbing Huang, Qile Hu, Changhua Lai

**Affiliations:** State Key Laboratory of Animal Nutrition, College of Animal Science and Technology, China Agricultural University, Beijing 100193, China; wanglucau@163.com (L.W.); q15928806587@163.com (L.W.); lzq2434390936@163.com (Z.L.); bbhuang217@163.com (B.H.); S20193040581@cau.edu.cn (Q.H.)

**Keywords:** endogenous loss, fat, fatty acids, vegetable oil, method, digestibility, growing pigs

## Abstract

**Simple Summary:**

Apparent digestibility of fat in diets does not reflect the true availability of fat, especially in low-fat diets. Estimation of endogenous losses of fat and fatty acids from the digestive tract is required for the determination of true digestibility of fat. This study evaluates the effect of oil sources on endogenous losses of fat and fatty acids in growing pigs in which endogenous losses were estimated by both regression and fat-free diet methods. Results indicate that the estimated values for endogenous losses of fat and fatty acids were not different in pigs fed palm oil, soybean oil, flaxseed oil or rapeseed oil. The fat-free diet had lower estimated values compared with the regression method. A fat-free diet method deserves to be explored further. These findings contribute to accurate estimation of endogenous losses of fat and fatty acids for vegetable oils in the future.

**Abstract:**

An experiment was conducted to determine the effect of oil sources with differing degrees of fatty acid saturation on endogenous losses of fat (ELF) and fatty acids (ELFA) in growing pigs, in which endogenous losses were estimated by two methods. Sixty-eight growing barrows (initial body weight 31.13 ± 4.44 kg) were randomly allotted to a completely randomized design with 17 diets. Sixteen added-oil diets were formulated by adding four levels (2%, 4%, 6% and 8%) of palm oil (PO), soybean oil (SBO), flaxseed oil (FSO) and rapeseed oil (RSO) to a diet poor in fat, respectively. One fat-free diet was formulated from cornstarch, soy protein isolate and sucrose. All diets contained chromic dioxide (0.4%) as an indigestible marker. Results indicated that, according to the regression equations, the amounts of ELF in PO, SBO, FSO and RSO were 6.28, 5.30, 4.17 and 4.84 g/kg of dry matter intake (DMI), respectively. The true total tract digestibility of fat was greater (*p* < 0.05) for FSO and RSO than for PO, and the ELFA were different from 0 only for C16:0, C18:0 and C18:1 in FSO, and C16:0 and C18:0 in RSO (*p* < 0.05). The estimated values for ELF and ELFAs in pigs fed PO, SBO, FSO or RSO were not different. The amount of ELF determined by the fat-free diet method was 2.60 g/kg DMI, and the amounts of C16:0, C18:0, C18:1 and C18:2 in ELFAs were 0.28, 0.26, 0.03 and 0.02 g/kg DMI, respectively. The fat-free diet method had lower ELF and ELFA values compared with the regression method (*p* < 0.01). Collectively, dietary vegetable oil sources do not affect estimation of ELF and ELFA, but different evaluation methods lead to varying estimates of endogenous losses in pigs.

## 1. Introduction

Endogenous fat is produced continuously from the digestive tract mainly including bile, desquamated epithelial cells, intestinal secretions and microbial lipids. These endogenous fats are mixed with dietary lipids then partially digested and absorbed. The unabsorbed fraction is excreted and considered as the endogenous loss of fat (ELF) [1,2]. Precise measurement of these inevitable losses is of great significance for the determination of true digestibility of fat and determining fatty acid requirements. True digestibility of fat is more reflective of fat availability [3]. Furthermore, fatty acids are the most important factors determining the nutritive value of fat [4,5]. However, there has been limited information about the endogenous losses of fatty acids (ELFA) published previously. 

ELF may be influenced by many factors. Previous studies have focused on comparing the ELF of intact oils with their corresponding extracted oils [6,7,8]. In addition, effects of dietary fiber on ELF have been well researched by Kil et al. [6] and Chen et al. [2]. Saturation degree and carbon chain length of fatty acids have an impact on digestion and absorption of oils in pigs [9], and thus may impact reabsorption of endogenous fat. However, data that compares ELF in different oil sources containing diverse fatty acid composition has been limited. Oils are often used in the swine industry to increase energy density in diets and to improve growth performance of swine [10]. Understanding the effect of different oils on ELF and ELFA systematically has important implications for effective utilization of oil in commercial practices.

Endogenous loss of fat can be measured by different methods. One of the most common methods is to provide graded dietary concentrations of fat and perform a regression to estimate endogenous loss and true digestibility of fat [11]. A second method requires feeding the animal a fat-free diet and measuring the fat content in feces, which represents the endogenous losses [2]. Regression methods have been widely used to evaluate ELF in pigs [6,7,8,11], but research on fat-free diet methods is limited. Comparing the two methods is important for future method selection.

One objective of this study was to determine the effect of oil sources with differing degrees of fatty acid saturation on endogenous losses of fat and fatty acids in growing pigs using palm oil (PO), soybean oil (SBO), flaxseed oil (FSO) and rapeseed oil (RSO). A second objective was to compare estimations of endogenous losses by fat-free diet and regression method.

## 2. Materials and Methods 

All protocols used in this experiment were reviewed and approved by the Institutional Animal Care and Use Committee of China Agricultural University (Beijing, China; No. AW12119102-1-1).

### 2.1. Animals and Housing

This study was conducted at FengNing Swine Research Unit of China Agricultural University (Academician Workstation in Chengdejiuyun Agricultural and Livestock Co., Ltd, Chengde, China). Sixty-eight growing barrows (Duroc × Landrace × Yorkshire; initial body weight 31.13 ± 4.44 kg) were housed individually in stainless-steel metabolism crates (1.4 × 0.7 × 0.6 m^3^). Barrows were fed the assigned test diets at 4% of initial body weight daily divided into two equal amount of meal at 09:00 and 16:00 h [12]. Water was available continuously for each pig. Room temperature was maintained at 22 ± 2 °C to meet the environmental needs of the pigs.

### 2.2. Experimental Diets

PO, SBO, FSO and RSO used in this experiment were food grade (free fatty acids < 0.2%). Fatty acid composition of the four oils were analyzed (Table 1). One diet was formulated from cornstarch, soy protein isolate and sucrose (fat-free diet), in which the fat content could be negligible. Sixteen diets were formulated by adding four levels (2%, 4%, 6% and 8%) of PO, SBO, FSO and RSO (Table 2), respectively. Four different levels of oils were included in diets at the expense of the energy ingredients of the fat-free diet. Added oils were the only source of fat and fatty acids. Rice hulls (5%) were included in the experimental diets as a fiber source. The same proportion of vitamins and minerals were supplemented to meet or exceed the nutrient requirements of growing pigs as recommended by the NRC (2012) [13]. Chromic oxide (Cr_2_O_3_; 0.40%) was included in all diets as an indigestible marker. 

### 2.3. Experimental Design and Sample Collection

Sixty-eight barrows were randomly allotted to a completely randomized design with 17 diets. Each diet was replicated four times with one pig per replicate. The experiment lasted 17 days, with the initial seven days used for cage adaptation, followed by seven days for diet adaptation. A sufficient adaptation was required before taking sampling of feces as low-fiber diets were used in the present experiment [14]. The last three days were used for feces collection.

Samples of diets and oils were collected and stored at 4 °C until analysis. Feces collection lasted 9 h daily beginning at 0800 h. During this period, fresh fecal samples were collected into plastic bags as soon as they appeared in the metabolism crates and were stored at −20 °C immediately [6]. At the end of the experiment, all feces samples were thawed and pooled, and a subsample was collected. Feces subsamples were lyophilized in a vacuum-freeze dryer (Tofflon Freezing Drying Systems, Minhang District, Shanghai, China) and ground through a 1-mm screen for further chemical analysis.

### 2.4. Chemical Analyses

Four oils, 17 diets and all fecal samples were analyzed for the content and type of fatty acids using Gas Chromatography (6890 Series, Agilent Technologies, Wilmington, DE, USA) according to the procedures of Sukhija and Palmquist [15] with the slight modification of using undecanoic acid (C11:0) (Sigma-Aldrich, St. Louis, MO, USA) as the internal standard. All diets and fecal samples in this experiment were analyzed for dry matter (DM; method 930.15) [16] and crude fat (ether extract (EE)) [17]. The chromium concentration in diets and feces were determined using a polarized Zeeman Atomic Absorption Spectrometer (Hitachi Z2000, Tokyo, Japan) after nitric acid-perchloric acid wet ash sample preparation. Seventeen diets were analyzed for crude protein (method 990.03) [16], acid detergent fiber (method 973.18) [16] and neutral detergent fiber [18].

### 2.5. Calculation

The ELF and true total tract digestibility (TTTD) of fat for the four oils were calculated using the regression method [11]. Apparently, digested fat (g/kg dry matter intake (DMI)) regressed against dietary fat intake (g/kg DMI) for each pig. The Y-intercept of this regression equation was considered the ELF (g/kg DMI), whereas the slope of the regression line represented true digestibility of fat [11]. The ELFA from the four sources of oil was determined using the same regression method. The contribution of ELF obtained by the regression method from total fecal fat (%) was calculated: contribution of ELF from total fecal fat (%) = 100 × concentration of ELF/(concentration of fat in feces × concentration of Cr_2_O_3_ in diet/concentration of Cr_2_O_3_ in feces). 

The ELF and ELFA over the entire intestinal tract also were determined in pigs fed fat-free diets (0.23% EE, DM basis) according to the following equation [19]: ELF or ELFA (g/kg DMI) = concentration of fat or fatty acids in feces × concentration of Cr_2_O_3_ in diet/concentration of Cr_2_O_3_ in feces.

The apparent total tract digestibility (ATTD; %) of fat in each added-oil diet was calculated using the indicator method [12]: ATTD (%) = 100 − 100 × (concentration of Cr_2_O_3_ in diet × concentration of fat in feces)/(concentration of Cr_2_O_3_ in feces × concentration of fat in diet). 

### 2.6. Statistical Analyses

Data were analyzed statistically using the GLM procedure of SAS (SAS Inst. Inc., Cary, NC, USA). An individual pig was the experiment unit for all analyses. The REG procedure of SAS was used to estimate the Y-intercept of the regression line for determining ELF or ELFA, and the slope was used to determine the TTTD of fat for oils. The intercept (ELF or ELFA) and slope (TTTD of fat) of regression equations were compared among the four sources of oil using the GLM procedure [20]. The relationship between fatty acid composition and ELF for each oil was determined using the CORR procedure. The *t*-test procedure was used to compare the ELF and ELFA obtained by fat-free diet and regression methods. Orthogonal polynomial contrasts were used to determine effects of increasing concentrations of oil on digestibility of fat. In all analyses, differences were considered significant if *p* < 0.05 and considered a trend if 0.05 < *p* < 0.10.

## 3. Results

### 3.1. Fatty Acid Composition for Four Oils 

There were dramatic differences in fatty acid composition among different oil sources (Table 1). The PO was rich in C16:0 and contained a large amount of saturated fatty acids (SFAs). The SBO, FSO and RSO were rich in C18:2, C18:3 and C18:1, respectively. The FSO and RSO contained a relatively high amount of unsaturated fatty acids (UFAs). The unsaturated to saturated fatty acids ratio (U:S) among the four oils ranged from 1.13 to 12.79.

### 3.2. Chemical Analysis of Experimental Diets

Chemical analysis of experimental diets is shown in Table 2. The purified ingredients (cornstarch and soy protein isolate) were included as the non-fat dietary components. The range of neutral detergent fiber content in experimental diets was from 5.10% to 6.62%. 

### 3.3. ELF and TTTD Obtained by the Regression Method 

The ELF and TTTD for four experimental oils were estimated by the regression method (Table 3). The total apparently digested fat over the entire intestinal tract linearly increased (*p* < 0.01, R^2^ > 0.97) with increased fat intake. The intercepts and slopes of four different oils were both significant (*p* < 0.01), as shown in regression equations. The estimated ELF values among PO, SBO, FSO and RSO diets ranged from 4.17 to 6.28 g/kg DMI. The amount of ELF did not differ among four different oils. The TTTD of fat was higher (*p* < 0.05) in FSO and RSO than in PO. 

### 3.4. Correlation between Fatty Acid Composition and ELF

There was no significant correlation between fatty acid composition and the amount of ELF obtained by the regression method, except that the amount of ELF had a tendency to be correlated positively (*p* < 0.10) with C16:0 and SFA (Table 4). 

### 3.5. ELFA Obtained by the Regression Method

The fatty acid composition of the four oils was primarily C16:0, C18:0, C18:1, C18:2 and C18:3; therefore, the ELFA for four oils was estimated by the regression method (Table 5), although the Y-intercepts were different from 0 only for C16:0, C18:0 and C18:1 in FSO, and C16:0 and C18:0 in RSO (0.79, 0.92, 0.14, 0.68 and 1.48 g/kg of DMI, respectively; *p* < 0.05). The estimated values for endogenous loss of individual fatty acid in pigs fed PO, SBO, FSO and RSO were not different.

### 3.6. ELF and ELFA Obtained by Fat-Free Diet Method

The results of the ELF and ELFA over the entire intestinal tract were obtained by the fat-free diet method (Table 6). The amount of ELF was 2.60 g/kg DMI. The major SFAs in ELFA were C15:0, C16:0, C18:0 and C24:0, whereas C18:1 and C18:2 were the main UFAs. These fatty acids collectively constituted 83% of total ELFA. The odd-chain fatty acids were also involved in ELFA, such as C15:0 and C17:0. The percentage of ELFA relative to the ELF was 37.69%. The fat-free diet method had lower ELF and ELFA values compared with the regression method (*p* < 0.01).

### 3.7. Apparent Digestibility of Fat and Contribution of ELF from Total Fecal Fat

As inclusion level of oil increased, ATTD of fat linearly increased regardless of oil sources (*p* < 0.01) and quadratically increased in pigs fed diets containing FSO (*p* < 0.01; Table 7). Furthermore, the contribution of ELF from total fecal fat decreased from 94.67% to 56.07% in PO, from 96.29% to 64.07% in SBO, from 93.09% to 67.76% in FSO and from 100.16% to 89.30% in RSO (Table 8).

## 4. Discussion

The regression method was used to estimate ELF and TTTD for four oils in the current study. A linear response of total apparently digested fat to dietary fat intake is a prerequisite of using the regression method [6,11]. The content and nature of fiber among diets should be similar for accurate estimation of the ELF [7]. These conditions were all met in the present experiment. In addition, this experiment utilized highly digestible, semi-purified diets to evaluate the fat excreted by pigs at zero fat intake, allowing for the estimation of ELF with little influence from dietary ingredients.

To our knowledge, this was the first study that estimated ELF value for FSO (4.17 g/kg DMI). In the current experiment, the ELF estimates for PO, SBO and RSO were 6.28, 5.30 and 4.84 g/kg DMI over the entire intestinal tract, respectively, which were less than 10.80 [21], 14.02 [22] and 23.0 g/kg DMI [8], respectively. This difference could be due to more purified ingredients in diets fed in the present study. Instead of feeding traditional diets based on natural ingredients (corn, barley, soybean meal, canola meal), cornstarch and soy protein isolate containing lower fiber content were formulated in the present diets. Previous studies reported that greater dietary fiber content resulted in increasing endogenous fat excretion [2,6,7]. In addition, purified ingredients with high digestibility may decrease endogenous fat excretion [22,23]. Estimation of ELF for SBO in this experiment was close to the value reported by Jørgensen et al. [11] because purified ingredients were used in both studies. Furthermore, the least concentration of dietary fat fed and range in fat concentration would affect the estimation of ELF [6].

Oil sources containing different fatty acid compositions may affect digestion, absorption and metabolic utilization of dietary fat [5], which may also affect reabsorption of endogenous fat. Therefore, four vegetable oils were chosen for their starkly different fatty acid compositions. When using the regression method, the estimated ELF values over the entire intestinal tract were not different in pigs fed PO, SBO, FSO or RSO. This observation is in agreement with a previous study where the amount of ELF was not different in pigs fed coconut oil or soybean oil [24]. Furthermore, there was no significant correlation between the concentration of ELF and fatty acid composition. This result confirms that vegetable oil sources containing diverse fatty acid composition had no effect on the estimation of ELF. The experiment indicates that the amount of ELF had a tendency (*p* < 0.10) to be positively correlated with SFAs. This may have been because fatty acid types could distinctly impact gut bacteria [25]. The polyunsaturated fatty acids had a stronger inhibitory effect on gut bacteria than SFAs [26], so the great amount of SFAs could have increased the excretion of bacterial lipids.

The TTTD of fat in pigs fed PO, SBO and RSO diets were very similar to those reported previously for PO (91.9%) [21], SBO (95.4%) [22] and RSO (100%) [8]. The TTTD of fat in diets was above 90%, regardless of oil sources, and reveals that vegetable oils are highly digestible ingredients for growing pigs. Within regression equations, greater TTTD of fat in pigs fed FSO and RSO diets rather than an PO diet was observed. The reason for this may be a higher U:S in FSO and RSO compared with PO [9]. 

To our knowledge, little information about ELFA of pigs exists. According to our regression equations (Table 6), average contents of endogenous losses of C16:0, C18:0 and C18:1 for four oils were 0.64, 0.81 and 0.11 g/kg DMI, respectively. These values were slightly higher than those of Jørgensen et al. [11], who reported that endogenous losses of C16:0, C18:0 and C18:1 for soybean oil were 0.41, 0.21 and 0.10 g/kg of DMI, respectively. In the study of Jørgensen et al. [11], however, addition of soybean oil was up to 3% only, which would have influenced the estimation of ELFA. The estimated values for endogenous loss of individual fatty acid in pigs fed PO, SBO, FSO or RSO were not different, indicating that the estimation of ELFA was not influenced by vegetable oil sources. In the current study, the fact that most of the estimated ELFA by the regression method was not different from 0 is a reflection of the relatively large variability in ELFA. 

A fat-free diet was fed to pigs to allow the measurement of fat content in feces that represented the ELF. However, unlike a protein-free diet, no routine fat-free diet was available for determining ELF in pigs. In this experiment, preparation of fat-free diets with cornstarch, soy protein isolate, sucrose and rice hull (0.23% EE, DM basis) resulted in an estimated value of 2.60 g/kg DMI for ELF. Chen et al. [2] researched the effect of fiber on ELFA in growing pigs using the fat-free diet method, and reported that the major components of ELFA over the entire intestinal tract were C16:0, C18:0, C18:1 and C18:2, which is in general agrees with our results. In addition to the even-chain fatty acids, bacteria also contain many odd-chain fatty acids [27,28]. The amounts of C15:0 and C17:0 in ELFA were relatively high in the current experiment, which confirms that microbial lipids could be a substantial source of ELF. Only 37.69% of the ELF was accounted for by ELFA in this experiment. The balance (62.31%) comes from non-fatty acid sources including cholesterol, bile acids [29], desquamated intestinal epithelial cells [1] or phospholipids and sphingolipids of microbial origin [1,30]. Similarly, Tancharoenrat et al. [31] reported that only 48.1% of the endogenous fat was of fatty-acid origin in broiler chickens. 

Notably, even under the closely controlled experimental conditions, differences in ELF and ELFA could be observed using different estimation methods. In the current experiment, the fat-free diet had lower ELF and ELFA values compared with the regression method. This was linked to the low fat content in the fat-free diet, which was far below the fat content of normal diets for growing pigs. The low fat content of the fat-free diet increases reabsorption of endogenous fat [1]. According to this experiment, we can clearly see that fewer pigs were required for the fat-free diet method. However, the regression method is more complicated and costly, and taking estimate values extrapolated to zero intake of a particular source of oil, instead of directly measuring fat content in feces, can bias the data, with the risk of overestimating the endogenous excretion. There is large variability in the estimation of ELFA by the regression method. Furthermore, the composition of the experimental diet used in the regression method has a large impact on endogenous losses, but no unified diets are available for determining ELF. Based on these considerations, estimation for endogenous losses using the fat-free diet method is an attractive method for correcting apparent digestibility of fat and fatty acids, and the fat-free diet deserves to be explored further.

The ATTD of fat in diet increased as the inclusion level of oil in diets increased, regardless of oil sources. This result is in agreement with previous studies in pigs fed extracted oil diets [6,7,11,21], intact oil diets [6,8,32] and diets containing both [33]. In this experiment, the contribution of ELF to total fecal fat decreased as the inclusion level of oil in diets increased, reflecting a greater effect of ELF on apparent digestibility at lower dietary fat levels than at higher levels. Furthermore, the amount of ELF obtained by the regression method would have accounted for more than 50% of the total fecal fat (Table 8). Therefore, the effect of ELF on the true digestibility of fat cannot be ignored. The ATTD of fat in diets could not reflect the true availability of fat, especially in low fat diets, so consideration should be given to estimating ELF to determine true digestibility.

## 5. Conclusions

The estimation of ELF and ELFA over the entire intestinal tract were not different when pigs were fed vegetable oils containing a diverse fatty acid composition, but different evaluation methods led to varying estimates of endogenous losses in pigs. The presence of C15:0 and C17:0 confirmed that microbial lipids could be a substantial source of ELF. The fat-free diet method had lower ELF and ELFA values compared with the regression method. Based on the practical considerations, a fat-free diet method deserves to be explored further. The present study suggested that the ATTD of fat is not additive, especially in low-fat diets and, therefore, fat digestibility values corrected for ELF should be used in diet formulation.

## Figures and Tables

**Table 1:** Fatty acid composition of four oil sources (% of total fatty acids).

Fatty Acid (FA)	Palm Oil (PO)	Soybean Oil (SBO)	Flaxseed Oil (FSO)	Rapeseed Oil (RSO)
C12:0	0.17	0.01	0.01	0.01
C14:0	0.95	0.08	0.04	0.05
C15:0	0.04	0.01	0.02	0.02
C16:0	40.96	11.08	5.16	4.03
C16:1	0.20	0.08	0.06	0.20
C17:0	0.09	0.01		0.09
C18:0	4.14	4.26	3.49	1.78
C18:1	41.99	23.16	17.82	61.51
C18:2	10.57	52.72	14.66	19.58
C18:3	0.17	7.24	57.87	9.91
C20:0	0.36	0.40	0.14	0.60
C20:1	0.14	0.20	0.22	1.23
C21:0	0.01	0.06	0.04	0.07
C20:3			0.02	
C20:4			0.02	
C20:3			0.05	
C22:0	0.07	0.46	0.13	0.33
C20:5				
C22:1	0.01	0.01	0.10	0.09
C22:2	0.04	0.02	0.02	0.06
C23:0	0.01	0.05	0.02	0.04
C24:0	0.08	0.15	0.09	0.22
C24:1			0.01	0.16
SFA	46.89	16.57	9.15	7.25
MUFA	42.34	23.44	18.21	63.19
PUFA	10.77	59.98	72.64	29.56
U:S	1.13	5.03	9.93	12.79

SFA, saturated fatty acid. MUFA, monounsaturated fatty acid. PUFA, polyunsaturated fatty acid. U:S, the ratio of unsaturated to saturated fatty acids.

**Table 2:** Ingredient composition (%, as-fed basis) and chemical analysis (%, DM basis) of experimental diets.

Items	Contents of Palm Oil, %				Contents of Soybean Oil, %				Conents of Flaxseed Oil, %				Contnets of Rapeseed Oil, %				Fat-Free
	2	4	6	8	2	4	6	8	2	4	6	8	2	4	6	8	
Ingredients																	
Cornstarch	60.34	58.98	57.62	56.26	60.34	58.98	57.62	56.26	60.34	58.98	57.62	56.26	60.34	58.98	57.62	56.26	61.70
Soy protein isolate	20.54	20.07	19.61	19.15	20.54	20.07	19.61	19.15	20.54	20.07	19.61	19.15	20.54	20.07	19.61	19.15	21.00
Palm oil	2.00	4.00	6.00	8.00													
Soybean oil					2.00	4.00	6.00	8.00									
Flaxseed oil									2.00	4.00	6.00	8.00					
Rapeseed oil													2.00	4.00	6.00	8.00	0.00
Sucrose	7.82	7.65	7.47	7.29	7.82	7.65	7.47	7.29	7.82	7.65	7.47	7.29	7.82	7.65	7.47	7.29	8.00
Rice hull	5.00	5.00	5.00	5.00	5.00	5.00	5.00	5.00	5.00	5.00	5.00	5.00	5.00	5.00	5.00	5.00	5.00
Dicalcium phosphate	2.50	2.50	2.50	2.50	2.50	2.50	2.50	2.50	2.50	2.50	2.50	2.50	2.50	2.50	2.50	2.50	2.50
Limestone	0.50	0.50	0.50	0.50	0.50	0.50	0.50	0.50	0.50	0.50	0.50	0.50	0.50	0.50	0.50	0.50	0.50
Sodium chloride	0.40	0.40	0.40	0.40	0.40	0.40	0.40	0.40	0.40	0.40	0.40	0.40	0.40	0.40	0.40	0.40	0.40
Chromic oxide	0.40	0.40	0.40	0.40	0.40	0.40	0.40	0.40	0.40	0.40	0.40	0.40	0.40	0.40	0.40	0.40	0.40
Vitamin-mineral premix ^1^	0.50	0.50	0.50	0.50	0.50	0.50	0.50	0.50	0.50	0.50	0.50	0.50	0.50	0.50	0.50	0.50	0.50
Analyzed composition ^2^																	
DM	90.07	90.82	90.08	90.60	89.84	90.53	90.46	90.97	90.28	90.52	89.89	91.39	90.03	90.59	90.23	90.75	89.99
EE	2.01	4.20	6.06	8.18	2.03	3.91	6.03	7.68	1.98	4.01	6.03	8.41	2.11	3.77	5.80	8.10	0.23
CP	20.60	19.95	19.07	17.56	20.94	19.15	18.91	18.67	20.30	19.56	19.24	18.04	17.88	21.23	18.92	17.27	19.58
NDF	6.07	6.62	6.36	6.53	5.20	5.94	5.23	5.81	5.91	5.10	6.17	5.68	6.24	5.61	5.48	5.16	5.84
ADF	2.53	2.95	3.21	3.34	3.12	3.10	3.30	3.01	2.98	2.63	3.06	2.88	3.05	3.39	2.95	2.79	3.35
Calculated composition ^3^																	
ME (MJ/kg)	14.60	14.93	15.26	15.60	14.48	15.08	15.48	15.89	14.68	15.08	15.48	15.89	14.66	15.05	15.44	15.83	14.27

^1^ Vitamin–mineral premix provided the following per kg of a complete diet for growing pigs: vitamin A, 5512 IU; vitamin D_3_, 2200 IU; vitamin E, 30 IU; vitamin K_3_, 2.2 mg; vitamin B_12_, 27.6 μg; riboflavin, 4.0 mg; pantothenic acid, 14.0 mg; niacin, 30.0 mg; choline chloride, 400.0 mg; folacin, 0.7 mg; thiamine 1.5 mg; pyridoxine 3.0 mg; biotin, 44.0 ug; Mn, 40.0 mg (MnO); Fe, 75.0 mg (FeSO_4_·H_2_O); Zn, 75.0 mg (ZnO); Cu, 100.0 mg (CuSO_4_·5H_2_O); I, 0.3 mg (KI); Se, 0.3 mg (Na_2_SeO_3_). ^2^ DM, dry matter; EE, ether extract; CP, crude protein; NDF, neutral detergent fiber; ADF, acid detergent fiber. ^3^ ME, metabolizable energy. Values were calculated from the Chinese Feed Database 2018.

**Table 3:** Endogenous loss of fat (ELF) over the entire intestinal tract and true total tract digestibility (TTTD) for palm oil, soybean oil, flaxseed oil and rapeseed oil obtained by the regression method ^1^.

Items	Regression Equation ^2^	R^2^	ELF (g/kg DMI) ^3^			TTTD (%) ^4^		
			Estimate	SE	*p*-Value	Estimate	SE	*p*-Value
Palm oil	y = 0.92x − 6.28	0.98	6.28	1.92	<0.01	92.37 ^b^	0.03	<0.01
Soybean oil	y = 0.95x − 5.30	0.98	5.30	1.69	<0.01	95.06 ^a,b^	0.03	<0.01
Flaxseed oil	y = 0.98x − 4.17	1.00	4.17	0.61	<0.01	97.60 ^a^	0.01	<0.01
Rapeseed oil	y = 0.99x − 4.84	0.99	4.84	1.20	<0.01	99.39 ^a^	0.02	<0.01

^a,b^ Within a column, values without a common superscript differ (*p* < 0.05). DMI, dry matter intake; SE, standard error. ^1^ Sixteen observations in each equation. ^2^ Calculated from regression equation y = ax + b, where x = dietary fat intake (g/kg of DMI), y = apparently digested fat (g/kg of DMI) of total intestinal tract. ^3^ For estimated ELF, the values among four oil sources were not different (*p* > 0.10 and standard error of the means = 1.97). ^4^ The TTTD of fat was higher in flaxseed oil and rapeseed oil than in palm oil (*p* = 0.03 and standard error of the means = 0.04).

**Table 4:** Correlation coefficients between fatty acid composition and estimated endogenous loss of fat (ELF) obtained by the regression method.

Fatty Acid	C16:0	C18:0	C18:1	C18:2	C18:3	SFA ^1^	MUFA ^2^	PUFA ^3^	U:S ^4^
ELF	0.91 *	0.55	0.30	0.03	0.84	0.91 *	−0.75	0.28	−0.85

* *p* < 0.10. ^1^ SFA, saturated fatty acid. ^2^ MUFA, monounsaturated fatty acid. ^3^ PUFA, polyunsaturated fatty acid. ^4^ U:S, the ratio of unsaturated to saturated fatty acids.

**Table 5:** The endogenous losses of fatty acids (ELFA, g/kg DMI) over the entire intestinal tract for palm oil, soybean oil, flaxseed oil and rapeseed oil obtained by the regression method ^1^.

Items	Regression Equation ^2^			R^2^	ELFA (g/kg DMI)		
					Estimate ^3^	SE	*p*-Value
Palm oil							
C16:0	y = 0.88x − 0.48			0.65	0.48	0.54	0.63
C18:0	y = 0.87x − 0.61			0.68	0.61	0.42	0.49
C18:1	y = 0.93x − 0.12			0.97	0.12	0.42	0.78
C18:2	y = 0.96x − 0.05			0.97	0.05	0.22	0.84
C18:3	y = 1.01x − 0.01			0.99	0.01	0.01	0.13
SFA	y = 0.89x − 1.12			0.99	1.12	0.41	0.56
UFA	y = 0.95x − 0.19			0.98	0.19	0.53	0.73
Soybean oil							
C16:0	y = 0.93x − 0.60			0.66	0.60	0.99	0.55
C18:0	y = 0.92x − 0.21			0.64	0.21	0.37	0.56
C18:1	y = 0.97x − 0.02			0.99	0.02	0.10	0.87
C18:2	y = 0.99x − 0.06			1.00	0.06	0.07	0.37
C18:3	y = 0.10x − 0.01			1.00	0.01	0.00	0.81
SFA	y = 0.95x − 0.93			0.55	0.93	1.49	0.55
UFA	y = 0.98x − 0.06			0.99	0.06	0.20	0.75
Flaxseed oil							
C16:0	y = 1.06x − 0.79			0.93	0.79	0.31	0.02
C18:0	y = 1.09x − 0.92			0.78	0.92	0.30	<0.01
C18:1	y = 0.99x − 0.14			0.99	0.14	0.06	0.03
C18:2	y = 1.00x − 0.09			1.00	0.09	0.06	0.20
C18:3	y = 0.10x − 0.01			1.00	0.01	0.01	0.37
SFA	y = 0.97x − 1.76			0.80	1.76	0.85	0.06
UFA	y = 0.99x − 0.28			1.00	0.28	0.12	0.03
Rapeseed oil							
C16:0	y = 1.04x − 0.68			0.98	0.68	0.17	<0.01
C18:0	y = 1.39x − 1.48			0.61	1.48	0.44	<0.01
C18:1	y = 0.99x − 0.16			1.00	0.16	0.08	0.07
C18:2	y = 0.10x − 0.06			1.00	0.06	0.03	0.06
C18:3	y = 0.10x − 0.01			1.00	0.01	0.01	0.34
SFA	y = 1.03x − 2.26			0.90	2.26	0.58	<0.01
UFA	y = 0.99x − 0.32			1.00	0.32	0.17	0.08

DMI, dry matter intake; SE, standard error; SFA, saturated fatty acid. UFA, unsaturated fatty acid. ^1^ Sixteen observations in each equation. ^2^ Calculated from regression equation y = ax + b, where x = dietary fat intake (g/kg of DMI), y = apparently digested fat (g/kg of DMI) of total intestinal tract. ^3^ Values for endogenous loss of individual fatty acid in pigs fed palm oil, soybean oil, flaxseed oil or rapeseed oil were compared, but no differences were observed (*p* > 0.10 and standard error of the means = 2.57).

**Table 6:** The endogenous losses of fat and fatty acids over the entire intestinal tract obtained by the fat-free diet method and regression method.

Items	Method		SEM	*p*-Value
	Fat-Free Diet (g/kg DMI)	Regression (g/kg DMI) ^1^		
Fat	2.60	5.15	0.02	<0.01
SFA				
C14:0	0.01			
C15:0	0.11			
C16:0	0.28	0.64	0.03	<0.01
C17:0	0.04			
C18:0	0.26	0.81	0.04	<0.01
C20:0	0.02			
C22:0	0.03			
C23:0	0.02			
C24:0	0.11			
UFA				
C18:1	0.03	0.11	0.01	<0.01
C18:2	0.02	0.07	0.01	<0.01
C18:3		0.01		
C24:1	0.01			
SFA	0.91	1.52	0.08	<0.01
MUFA	0.05			
PUFA	0.02			
UFA	0.07	0.21	0.02	<0.01
TFA	0.98	1.73	0.02	<0.01

DMI, dry matter intake; SEM, standard error of the means; SFA, saturated fatty acid; MUFA, monounsaturated fatty acid; PUFA, polyunsaturated fatty acid; UFA, unsaturated fatty acids; TFA, total fatty acids. ^1^ Average content of endogenous losses of fat and fatty acids for four oils.

**Table 7:** Apparent total tract digestibility (%) of fat in experimental diets.

Items	Inclusion Level (%)				SEM	*p*-Value	
	2	4	6	8		Linear	Quadratic
Palm oil	65.02	74.74	79.36	86.56	3.45	<0.01	0.72
Soybean oil	64.65	72.20	86.77	87.89	4.38	<0.01	0.42
Flaxseed oil	68.45	83.37	91.23	92.25	1.77	<0.01	<0.01
Rapeseed oil	80.88	82.14	92.85	93.46	2.55	<0.01	0.99

SEM, standard error of the means.

**Table 8:** The contribution of endogenous loss of fat (%) obtained by regression method from total fecal fat in experimental diets.

Items	Inclusion Level (%)			
	2	4	6	8
Palm oil	94.67	63.88	58.14	56.07
Soybean oil	96.29	84.30	77.66	64.07
Flaxseed oil	93.09	82.43	70.48	67.76
engRapeseed oil	100.16	96.54	91.38	89.30
